# Case Report: Esophageal balloon occlusion with a Foley catheter in high-risk sedated gastroscopy

**DOI:** 10.3389/fmed.2026.1766538

**Published:** 2026-02-25

**Authors:** Jun Hu, Fenfen Kou, Peng Jiang, Ping Zhao, Yanhua Luo, Bao Lang, Shaojie Zhang

**Affiliations:** 1 Department of Anesthesiology, Weifang People's Hospital, Weifang, China; 2 Department of Gynecology, Affiliated Hospital of Shandong Second Medical University, Weifang, China; 3 Department of Gastroenterology, Weifang People's Hospital, Weifang, China

**Keywords:** airway management, aspiration prevention, case report, esophageal balloon occlusion, Foley catheter, post-gastrectomy, sedation

## Abstract

**Background:**

Patients with altered upper gastrointestinal anatomy (such as after proximal gastrectomy) face a significantly elevated risk of gastro-esophageal reflux and pulmonary aspiration during sedated endoscopy. There is a need for safe sedation strategies in these high-risk cases.

**Case presentation:**

We describe a 56-year-old male with a history of proximal gastrectomy and chronic reflux who required an upper endoscopy under sedation. To mitigate aspiration risk, an 18-Fr Foley catheter with an inflatable cuff was inserted transnasally into the mid-esophagus under light sedation and local anesthesia, and the balloon was inflated with 20 ml saline to occlude the esophageal lumen. The catheter's drainage port was connected to wall suction at a negative pressure of approximately −20KPa to aspirate any refluxate below the occlusion. After deepening sedation (MOAA/S score ≤ 2) with propofol and alfentanil (without endotracheal intubation), the endoscopy was performed while the esophageal balloon was gradually deflated under direct visualization. No obvious reflux or escape of gastric contents was observed during controlled deflation. The 8-min procedure was completed without hypoxemia, coughing, or any signs of aspiration, and the patient recovered without complications.

**Conclusion:**

This case suggests that the use of esophageal balloon occlusion with negative-pressure suction was associated with safe, uneventful deep sedation in a patient at high risk of aspiration. The technique may serve as a simple, minimally invasive alternative to endotracheal intubation or awake endoscopy for airway protection in such high-risk patients, though further evaluation in larger studies is warranted.

## Introduction

Regurgitation of gastric contents and subsequent pulmonary aspiration remain recognized complications of upper endoscopy performed under sedation or anesthesia (1–4). These risks are accentuated in patients with altered upper gastrointestinal anatomy—particularly those who have undergone total or proximal gastrectomy—because surgery disrupts the lower esophageal sphincter and the angle of His, facilitating reflux (5–7). Although strict fasting reduces gastric volume, aspiration cannot be completely eliminated; residual gastric contents are reported in up to 19% of apparently fasted patients (8).

Several strategies have been proposed to manage high aspiration risk during endoscopy. Performing the examination while the patient is fully awake preserves airway reflexes but is poorly tolerated and may itself provoke vomiting and reflux (9, 10). General anesthesia with endotracheal intubation provides reliable airway protection but requires neuromuscular blockade, specialized equipment and recovery resources, making it disproportionate for routine diagnostic gastroscopy (11). Various supraglottic airway devices (e.g., LMA^®^ Gastro™) permit deep sedation and airway management but do not prevent reflux (12). The Sengstaken–Blakemore tube, which incorporates both gastric and esophageal balloons, is primarily used to control variceal hemorrhage (13). Its esophageal balloon concept inspired our approach of temporarily occluding the esophageal lumen; however, the device's indications and hardware are not geared toward aspiration prevention during routine gastroscopy (14–16).

To our knowledge, esophageal balloon occlusion with continuous suction to enable sedated gastroscopy in post-gastrectomy patients has not previously been reported. Here we present a case in which this technique was used as an intermediate option between awake endoscopy and general anesthesia. We emphasize procedural details, placement confirmation, suction parameters and sedation targets to facilitate reproducibility and critically discuss its limitations and place in the continuum of airway-protective strategies.

## Case presentation

### Patient information and pre-procedure preparation

A 56-year-old man (172 cm, 60 kg, ASA II) presented for routine surveillance gastroscopy 10 years after proximal gastrectomy for cardia cancer. He complained of frequent acid reflux and heartburn but had no other major comorbidities. Pre-procedure evaluation identified him as extremely high risk for aspiration because of altered anatomy and chronic gastro-esophageal reflux. After discussing options (awake endoscopy, general anesthesia with intubation, or alternative techniques), he consented to attempted esophageal balloon occlusion under sedation. He fasted for 8 h prior to the procedure.

On arrival in the endoscopy suite, standard monitors were applied (continuous electrocardiography, non-invasive blood pressure, pulse oximetry) and oxygen was administered via nasal cannula at 4 L/min. Light sedation with midazolam 1 mg IV and topical nasal anesthesia with 3 ml of 2% lidocaine were given to reduce anxiety and facilitate catheter insertion.

### Catheter placement and sedation

**Figure 1:**
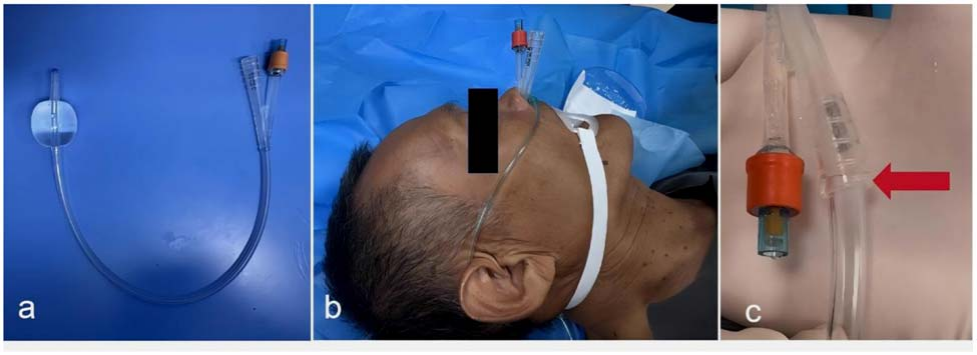
Key steps of the esophageal balloon occlusion technique. **(a)** The cuffed silicone catheter is filled with 20 ml of normal saline; **(b)** Prior to the induction of anesthesia, the catheter is inserted transnasally under topical nasal anesthesia and light sedation; **(c)** The catheter's drainage port is connected to a wall suction device.

Under light sedation and topical anesthesia, an 18-Fr silicone Foley catheter (double-lumen with an inflatable balloon) was inserted transnasally. External measurement from the nares to the xiphoid process (approximately 30–35 cm) guided depth and helped estimate placement in the mid-esophagus. The absence of coughing or dyspnoea during insertion suggested esophageal rather than tracheal placement. The balloon was gradually inflated with 20 ml of sterile saline while monitoring for respiratory distress; inflation created a temporary seal within the esophagus. The catheter's drainage port was connected immediately to continuous wall suction, maintained at approximately −20 KPa, to aspirate any fluid accumulating below the balloon and decompress the remnant stomach (Figure 1).

After confirming satisfactory placement, deep sedation was induced with intravenous alfentanil (~4 μg/kg) and propofol (~1.5 mg/kg) titrated to achieve a Modified Observer's Assessment of Alertness/Sedation (MOAA/S) score ≤ 2 while preserving spontaneous respiration. No neuromuscular blocking agents were used and endotracheal intubation equipment remained available in case of failure or complications.

### Endoscopic procedure and recovery

**Figure 2:**
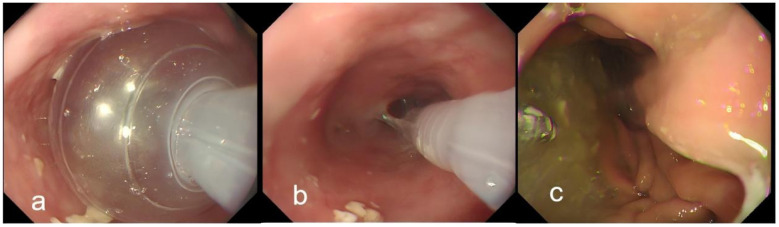
Intra-procedural endoscopic views during balloon occlusion. **(a)** The inflated balloon cuff completely occludes the esophageal lumen, preventing refluxate from ascending; **(b)** The balloon is gradually deflated under direct endoscopic visualization, allowing careful inspection for any reflux fluid; **(c)** Upon advancing the endoscope past the deflated balloon, a large amount of retained gastric content is observed in the stomach. A complete demonstration of this procedure is available in Supplementary Video1.

Once deep sedation was achieved, an upper endoscope was passed orally. Under direct endoscopic visualization, the balloon was partially and then completely deflated over several minutes while suction was maintained. The team monitored carefully for any evidence of reflux; had fluid been observed, the balloon would have been re-inflated and the regurgitated material aspirated before proceeding. After confirming the absence of visible reflux, the endoscope was advanced to examine the gastric remnant and anastomosis. Although retained gastric contents were present, they did not reflux into the esophagus(Figure 2). The entire procedure, from endoscope insertion to removal, lasted approximately 8 min; total balloon occlusion time was about 4 min. Throughout, the patient remained hemodynamically stable (blood pressure and heart rate within 20% of baseline; SpO_2_ ≥ 98%), without coughing, retching or desaturation.

Upon arrival in the post-anesthesia care unit, he regained full consciousness within 6 min. He reported only mild transient nasal discomfort and no sore throat or respiratory symptoms. He was discharged home later that day without complications.

### Procedural timeline

**Figure 3:**
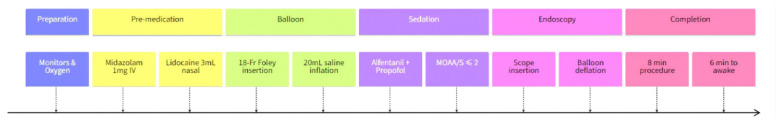
Procedural timeline.

To visualize the chronological sequence of events, Figure 3 illustrates the key time points from fasting through recovery.

### Patient perspective

The patient understood that his altered anatomy placed him at high risk of aspiration with standard sedation and expressed anxiety about undergoing an unsedated procedure or general anesthesia. He agreed to the esophageal balloon technique and afterwards reported that the experience was more comfortable than anticipated: he had no memory of the procedure, experienced only brief nasal irritation, and appreciated avoiding intubation.

## Discussion

This case illustrates a novel application of a commonly available device—a Foley urinary catheter—to mitigate aspiration risk during deeply sedated gastroscopy. By creating a controllable barrier within the esophagus and applying continuous suction, the technique allowed sedation without endotracheal intubation while permitting the endoscopist to control reflux dynamically.

Pulmonary aspiration remains a recognized complication of upper endoscopy even when fasting guidelines are followed, particularly in patients with altered anatomy or severe gastro-esophageal reflux (8, 17–19). Awake, unsedated examinations preserve airway reflexes yet are often intolerable and may provoke gagging or vomiting (9, 10). Routine prophylactic endotracheal intubation secures the airway but requires general anesthesia, neuromuscular blockade and prolonged recovery and carries risks of pharyngeal injury and ventilator-associated pneumonia (20–23). Specialized supraglottic devices such as the LMA^®^ Gastro™ facilitate deep sedation but do not prevent reflux (12). Our case therefore illustrates an intermediate strategy that balances patient comfort, procedural stimulation and airway protection through transnasal esophageal balloon occlusion with continuous suction. By creating a dynamic barrier at the source of reflux, the technique allowed deep sedation without intubation and may complement existing airway-management approaches for selected high-risk individuals.

The concept for this technique was inspired by the Sengstaken—Blakemore tube, a three-lumen two-balloon device used for gastro-esophageal variceal bleeding (15, 16). To actively control reflux in our case, we repurposed a standard 18-Fr silicone Foley catheter. The catheter is approximately 40 cm in total length, with the bifurcation located about 35 cm from its rounded tip; when inserted fully through the nostril, the tip rests in the mid-esophagus while the bifurcation remains at the nares. After measuring the distance from the nares to the xiphoid process to estimate esophageal insertion depth, we inflated the balloon with 20 ml of saline. In *ex vivo* measurements this inflation produced a diameter of roughly 3 cm, which approximates the upper range of the normal adult esophagus (~20–30 mm) (24)and is slightly larger than the inner diameters of most self-expanding esophageal stents (17–23 mm) (25, 26) or esophageal dilation balloons inflated with 50 ml (24–29 mm) (27). A 3 cm diameter balloon can occlude the esophageal lumen temporarily without excessive distention, while continuous suction through the drainage lumen decompresses the stomach and removes any refluxate.

Under light sedation, the patient was able to report discomfort, aiding confirmation that the catheter was in the esophagus rather than the trachea; after verifying correct placement, sedation was deepened and the balloon deflated under endoscopic visualization. If reflux were observed, the balloon could be reinflated to aspirate gastric contents before continuing. Total occlusion time was limited to about 4 min. Although guidelines for esophageal tamponade devices recommend deflating balloons every 2–8 h and removing them within 24 h to minimize mucosal necrosis (14, 16, 28), our occlusion was far shorter; should longer occlusion be necessary, we suggest limiting inflation to 10–15min, intermittently deflating the balloon and monitoring for mucosal ischemia. Future applications of this technique will incorporate bedside ultrasound and end-tidal carbon dioxide monitoring to confirm esophageal placement and avoid inadvertent tracheal insertion.

The limitations and risks of this method must be considered carefully. This report describes a single patient and cannot establish causality; the absence of aspiration may reflect the balloon, the suction, patient anatomy or chance. Transnasal insertion of a relatively large-bore catheter may be uncomfortable, and overinflation or prolonged inflation could injure the esophageal mucosa. The procedure demands close coordination between the anesthesiologist and endoscopist, and emergency airway equipment must be immediately available. Accordingly, the technique should only be attempted in centers with experienced teams and is most suitable for elective diagnostic examinations in patients at high risk of aspiration, such as those after gastrectomy, refractory gastro-esophageal reflux or severe gastric emptying disorders. It is contraindicated in suspected foreign-body impaction, strictures, varices or active bleeding. Importantly, acute airway obstruction from an impacted foreign body lodged in the upper esophagus represents a distinct scenario: such impaction can compress the posterior tracheal wall and cause rapid respiratory failure, necessitating emergent intubation and removal (29). Recognizing this continuum—from chronic aspiration risk to acute obstruction—helps clinicians select the appropriate airway-protection strategy.

Beyond its potential role in airway management, our case also exemplifies the creative repurposing of a common and inexpensive device to address a specific clinical challenge. A standard 18-Fr silicone Foley catheter served both as an occlusion balloon and a suction conduit, demonstrating that existing tools can be applied flexibly to manage complex situations. Similar inventive uses of Foley catheters have been reported in other settings—for example, using the catheter as a temporary traction device during laparoscopic surgery (30). Identifying such parallels encourages clinicians to leverage familiar equipment in novel ways and underscores the importance of innovation in resource-limited scenarios.

Transnasal esophageal balloon occlusion with suction may therefore offer a pragmatic compromise between awake endoscopy and general anesthesia with intubation. By establishing a temporary barrier and enabling dynamic control of reflux, the technique allowed us to perform sedated gastroscopy safely in a patient with extreme aspiration risk. Further cases and controlled studies are required to validate its safety, refine technical parameters (balloon size, inflation volume and duration) and define its role within the spectrum of perioperative airway management.

## Conclusion

In this post-gastrectomy patient at extreme aspiration risk, transnasal esophageal balloon occlusion with continuous suction permitted deep sedation without endotracheal intubation and was associated with an uneventful endoscopic procedure. By creating a temporary barrier and actively controlling reflux, the technique may offer a pragmatic bridge between awake endoscopy and general anesthesia for selected high-risk individuals. Nevertheless, its use should be regarded as exploratory: evidence is limited to a single case, the potential for mucosal injury during longer procedures is unknown, and it is contraindicated in patients with esophageal lesions or foreign bodies. Additional cases and controlled studies are required to clarify its safety, efficacy and appropriate indications.

## Data Availability

The raw data supporting the conclusions of this article will be made available by the authors, without undue reservation.
